# Monthly Record of Deaths in the City of Buffalo

**Published:** 1858-12

**Authors:** H. D. Garvin

**Affiliations:** Health Physician


ART. XI.—*Report of Mortality in Buffalo for the Month of Oct.*, 1858. By H. D. Garvin, M. D., Health Physician.

| DISEASES. | No. | Males. | Females. | No Sex given. |
|---|---|---|---|---|
| Accident, | 6 | 5 | 1 | |
| Albuminuria, | 1 | 1 | | |
| Angina Maligna, | 1 | 1 | | |
| Apoplexy, | 2 | 2 | | |
| Ascites, | 1 | | 1 | |
| Atrophia, | 1 | 1 | | |
| Cancer of Breast, | 2 | | 2 | |
| Cholera Infantum, | 5 | 4 | 1 | 1 |
| Cholera Morbus, | 1 | 1 | | |
| Congestion of Brain, | 1 | 1 | | |
| " " Lungs, | 1 | 1 | | |
| Convulsions, | 11 | 6 | 4 | 1 |
| Croup, | 2 | | 1 | 1 |
| Dentition, | 4 | 1 | 3 | |
| Diarrhœa, | 2 | 1 | | 1 |

### REGISTER OF MORTALITY—Continued.

| DISEASES. | No. | Males. | Females. | No Sex given. |
|---|---|---|---|---|
| Disease of Bowels, | 1 | | 1 | |
| " Lungs, | 1 | 1 | | |
| " Heart, | 1 | | 1 | |
| Dysentery, | 4 | 2 | 2 | |
| Enteritis, | 1 | 1 | 1 | |
| Fever, Typhoid, | 5 | 4 | 1 | |
| Gastritis, | 3 | 1 | 2 | |
| Gastro-Enteritis, | 1 | | 1 | |
| Gangrene of Mouth, | 1 | 1 | | |
| Hooping Cough, | 3 | 2 | 1 | |
| Hydrocephalus, | 2 | | 2 | |
| Intemperance, | 4 | 2 | 2 | |
| Jaundice, | 1 | | 1 | |
| Marasmus, | 10 | 7 | 2 | 1 |
| Old Age, | 4 | 1 | 3 | |
| Ossification of Heart, | 1 | 1 | | |
| Paralysis, | 3 | 3 | | |
| Phthisis, | 13 | 6 | 7 | |
| Premature Birth, | 1 | | | 1 |
| Rupture of Blood-Vessel, | 1 | 1 | | |
| Rupture of Uterus, | 1 | | 1 | |
| Spinal Disease, | 1 | | 1 | |
| Still Born, | 8 | 5 | 3 | |
| Small Pox, | 3 | 2 | 1 | |
| Tabes Mesenterica, | 1 | 1 | | |
| Thrush, | 1 | | 1 | |
| Unknown, | 8 | | | |
| Total, | 126 | | | |

### SEXES.

| | |
|---|---|
| Males, | 66 |
| Females, | 47 |
| Sex not given, | 6 |
| Total, | 126 |

### AGES.

| | | | |
|---|---|---|---|
| Still-born, | 8 | Between 20 years and 30 years, | 6 |
| 1 day, | 0 | " 30 " " 40 " | 11 |
| 1 day and 30 days, | 5 | " 40 " " 50 " | 16 |
| Between 1 month and 6 months, | 15 | " 50 " " 60 " | 5 |
| " 6 months and 12 " | 11 | " 60 " " 70 " | 2 |
| " 1 year " 3 years, | 23 | " 70 " " 80 " | 4 |
| " 3 " " 5 " | 9 | " 80 " " 90 " | 1 |
| " 5 " " 10 " | 2 | " 90 " " 100 " | 1 |
| " 10 " " 20 " | 3 | " 100 " | 0 |
| | 76 | | 86 |
| | | | 76 |

| | |
|---|---|
| Ages not given, | 4 |
| Total, | 126 |

### NATIVITIES.

| | | | |
|---|---|---|---|
| American, | 87 | English, | 6 |
| German, | 17 | Scotch, | 1 |
| Irish, | 15 | | — |
| Total, | | | 126 |

